# Advanced tumour-induced osteomalacia secondary to sinonasal phosphaturic mesenchymal tumour

**DOI:** 10.4102/sajr.v28i1.2975

**Published:** 2024-10-22

**Authors:** Monica van Wijk, Leon Janse van Rensburg, Bianca D. Berndorfler, Johan F. Opperman, Johan Grobbelaar, Amir H. Afrogheh, Sarah Versveld, Razaan Davis

**Affiliations:** 1 Division of Radiodiagnosis, Department of Medical Imaging and Clinical Oncology, Faculty of Medicine and Health Sciences, Stellenbosch University and Tygerberg Hospital, Cape Town, South Africa; 2 Nuclear Medicine Division, Department of Medical Imaging and Clinical Oncology, Faculty of Medicine and Health Sciences, Stellenbosch University and Tygerberg Hospital, Cape Town, South Africa; 3 Department of Oral and Maxillofacial Pathology, National Health Laboratory Service and University of the Western Cape, Cape Town, South Africa; 4 Division of Anatomical Pathology, Faculty of Medicine and Health Sciences, Stellenbosch University, Cape Town, South Africa; 5 Division of Otorhinolaryngology, Department of Surgery, Faculty of Medicine and Health Sciences, Stellenbosch University and Tygerberg Hospital, Cape Town, South Africa; 6 Division of Endocrinology, Department of Internal Medicine, Faculty of Medicine and Health Sciences, Stellenbosch University and Tygerberg Hospital, Cape Town, South Africa

**Keywords:** phosphaturic, mesenchymal, tumour, induced, osteomalacia, TIO, fractures, sinosanal, radiology, nuclear

## Abstract

Phosphaturic mesenchymal tumours (PMT) are rare, benign lesions and the most common tumour to cause tumour-induced osteomalacia (TIO), a paraneoplastic syndrome. Patients frequently exhibit severe osteomalacia, accompanied by multiple fractures, which significantly impair their quality of life. Complete surgical resection is curative.

**Contribution:**

A rare case of sinonasal PMT is presented, with a focus on the imaging findings and role of the radiologist and nuclear physician.

## Introduction

Phosphaturic mesenchymal tumours (PMT) are usually benign tumours that can arise from any soft tissue or osseous location.^1^ The majority of tumours are located in the skeleton and soft tissues of the extremities with only approximately 5% found in the head and neck. Reported head and neck sites include the paranasal sinuses, mandible, nasal cavity as well as the skull base. Phosphaturic mesenchymal tumours are by far the most common tumour to cause tumour-induced osteomalacia (TIO), a rare paraneoplastic syndrome due to tumoural release of fibroblast growth factor (FGF)-23 causing phosphate wasting; clinically presenting with fatigue, bone pain, fractures, and a resistance to vitamin D supplementation. Complete surgical resection is curative.^2,3,4,5,6^

## Patient presentation

A 56-year-old female presented multiple times to various healthcare providers over a period of 7 years, with a complaint of progressively worsening generalised body pain and weakness, since the age of 48 years. She also reported a constantly blocked nose with occasional moderate epistaxis. She was managed with analgesic medication, decongestants and antidepressants. Her condition had a debilitating impact on her physical abilities, her work and family life. She had numerous falls and was eventually wheelchair bound because of the incapacitating pain. Radiographs were eventually obtained by a primary healthcare provider and she was subsequently referred to a tertiary institution.

## Ethical considerations

Written informed consent was obtained from the patient. Ethics approval was granted by Stellenbosch University Health Research Ethics Committee (HREC) with reference number C24/02/005 on 07 March 2024.

## Management and outcome

### Radiographs

**FIGURE 1:**
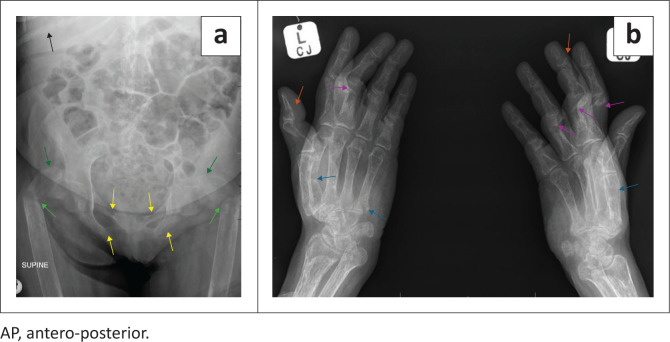
(a) AP radiograph of the pelvis: Generalised loss of bone mineral density. Cranially displaced subcapital as well as transverse sub trochanteric femur fractures (green arrows). Bilateral superior and inferior pubic rami fractures (yellow arrows). Right posterior 10th rib fracture (black arrow). (b) Oblique radiographs of bilateral hands: Generalised loss of bone mineral density. Multiple bilateral proximal phalanx fractures with dorsal angulation (purple arrows). Bilateral distal phalanx fractures (orange arrows). Bilateral metacarpal fractures (blue arrows).

Pelvic radiographs demonstrated diffuse loss of bone mineral. Bilateral comminuted proximal femoral fractures, with cranially displaced subcapital as well as transverse sub-trochanteric fractures, were present. Bilateral inferior and superior pubic rami fractures were observed. The imaged lower ribs demonstrated a right posterior 10th rib fracture (Figure 1a).

Radiographs of the hands showed marked diffuse osteomalacia with cortical thinning and prominent trabeculae. There were bilateral metacarpal and phalangeal fractures with dorsal angulation of the proximal phalanx fractures. No acro-osteolysis was present and there was no subperiosteal resorption (Figure 1b).

### Biochemistry

Reduced inorganic phosphate levels of 0.57 mmol/L (0.78–1.42); raised alkaline phosphatase levels of 420 U/L (42–98) and normal parathyroid hormone levels of 5.1 pmol/L (1.5–7.6) were seen. These initial biochemical results were obtained during one of her numerous health facility visits, but seemingly not recognised as TIO at the time. Further workup at the tertiary institution revealed a profound, isolated hypophosphatemia, with a raised alkaline phosphatase (ALP). Urine confirmed urine-phosphate losses with a fractional excretion of 56%, as well as a decreased tubular reabsorption of phosphate. Serum parathyroid hormone (PTH) was normal, as was the serum calcium, which ruled out parathyroid pathology. There were no other abnormalities found on urine analysis: urine calcium, bicarbonate, uric acid, protein and glucose were all negative, which ruled out an intrinsic renal tubular abnormality as the cause of her phosphate wasting. This led to the conclusion that the hypophosphatemia was secondary to fibroblast growth factor 23 (FGF-23) abnormalities.

### Advanced imaging

**FIGURE 2:**
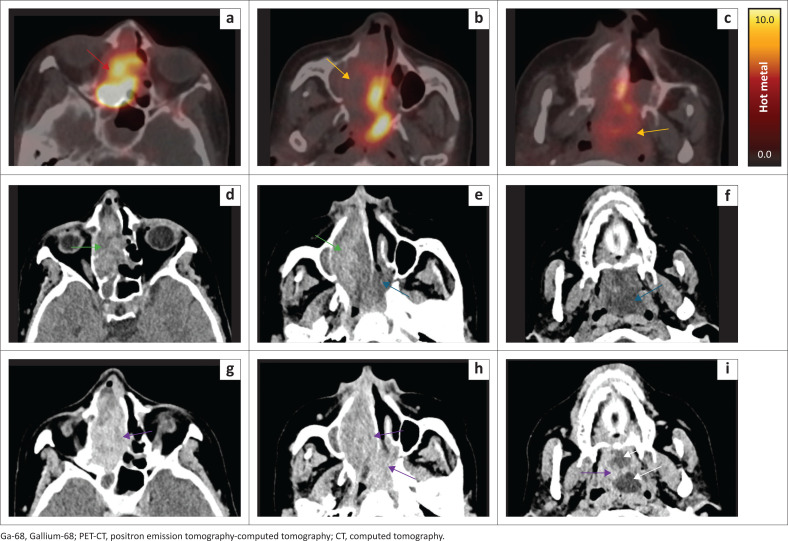
(a–c) Ga-68 DOTANOC PET-CT (d–f) Uncontrasted CT (g–i) Post-contrast CT. Multistudy axial imaging through the paranasal sinuses from superior to inferior, at corresponding levels, showing a soft tissue mass in the right ethmoid sinuses and nasal cavity extending posteriorly into the oro-nasopharynx. (a–c) Intensely DOTANOC avid mass (red arrow) with photopaenic lateral nasal passage and nasopharyngeal components (orange arrows). (d–f) Heterogenous appearance on uncontrasted CT with both hyper- (green arrows) and hypo- (blue arrows) attenuating components. (g–i) Post-contrast enhancement (purple arrows) with residual areas of hypoattenuation in the nasopharynx (white arrows).

Biochemical and radiographic findings suggested that an FGF-23-secreting tumour was present. Clinical examination failed to localise the tumour. An axillary dermal nodule was found and may have been misleading. This lesion was biopsied; however, histology showed a nonspecific inflammatory process. Whole body ^68^Gallium-DOTANOC PET-CT was then performed as first-line investigation for an occult lesion. A right-sided sinonasal mass demonstrated intense heterogeneous DOTANOC uptake. Some areas of soft tissue in the lateral nasal passage and nasopharynx were photopaenic. On the accompanying low-dose uncontrasted CT, this correlated with a soft tissue mass in the right ethmoid sinuses and nasal cavity extending posteriorly into the oro-nasopharynx (Figure 2a–c).

A multiplanar reconstructed diagnostic CT demonstrated a heterogeneous sinonasal mass centred in the right posterior ethmoid air cells and extending into the nasal passage and oro-nasopharynx. There were regions of internal soft tissue hyperdensity on uncontrasted CT, but no internal osseous or mineralised matrix. The serpiginous hyperdensities in the lateral aspect of the nasal soft tissue corresponded to the non-avid component on functional imaging. The lesion enhanced heterogeneously after contrast administration. Low-density fluid with rim enhancement was noticed in the polypoid nasopharyngeal component (Figure 2d–f).

**FIGURE 3:**
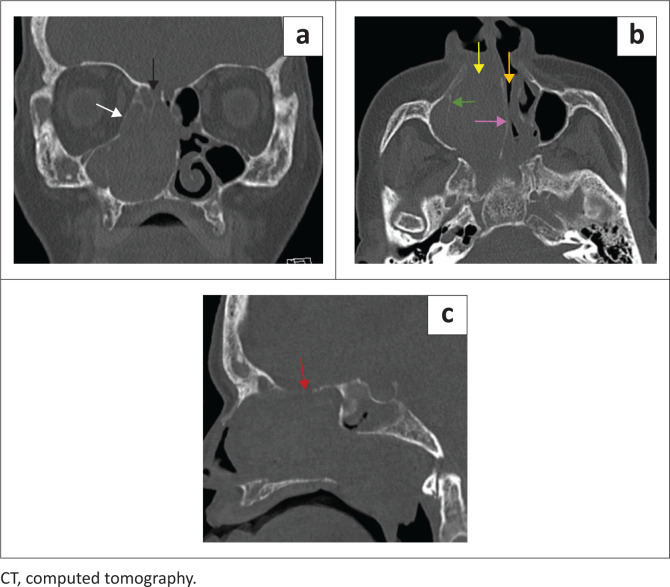
Bony window coronal (a), axial (b) and sagittal (c) CT images demonstrating displacement, remodelling and dehiscence of the right cribriform plate (black arrow), planum sphenoidale (red arrow), lamina papyracea (white arrow), medial maxillary sinus wall (green arrow) and nasal septum (purple arrow). Soft tissue mass obstructing the right nasal passage (yellow arrow). Displacement of the bony nasal septum to the left, obstructing the left nasal passage (orange arrow).

The mass was expansile, with remodelling of the sinuses and widening the right nasal passage, displacing the nasal septum and causing bilateral nasal passage obstruction. Osseous dehiscence of the lateral wall of the right maxillary sinus, the right lamina papyracea, the right cribriform plate and the planum sphenoidale was present (Figure 3).

**FIGURE 4:**
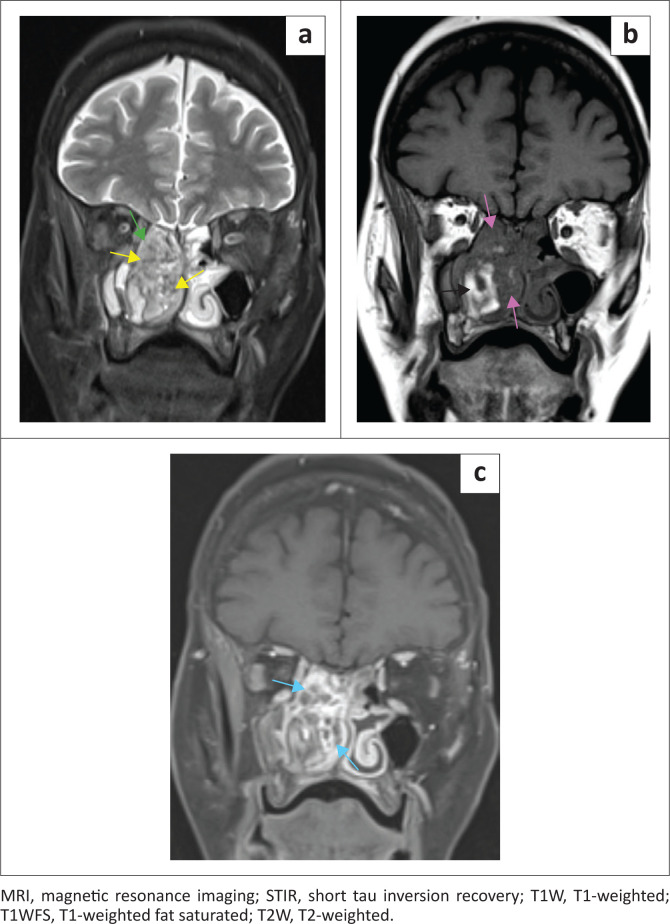
Coronal MRI STIR (a), T1W (b) and T1WFS post contrast (c). (a) Sinonasal tumour is T2W heterogeneous and hyperintense (green arrow) with T2W dark flow voids (yellow arrows). (b) T1W isointense to muscle (purple arrows) with a lateral hyperintense component (black arrow). (c) Heterogeneous post-contrast enhancement (blue arrows).

On MRI, the lesion was hyperintense and heterogeneous on fat saturated T2-weighted (T2W) and short tau inversion recovery (STIR), respectively, and contained T2W flow voids in keeping with a hypervascular lesion. The mass was predominantly isointense to muscle on T1-weighted (T1W), with avid and heterogeneous post-contrast enhancement. The CT hyperdense and DOTANOC non-avid component in the right lateral aspect of the tumour corresponded to serpiginous T1W, T2W and STIR hyperintense signal with low ADC values and high DWI values (mimicking restricted diffusion), consistent with subacute intratumoural haemorrhage. Within the polypoid nasopharyngeal tumour, the presence of internal T2W hyperintense signal and T1W isointense signal indicated chronic intratumoural haemorrhage (Figure 4 – Figure 6).

**FIGURE 5:**
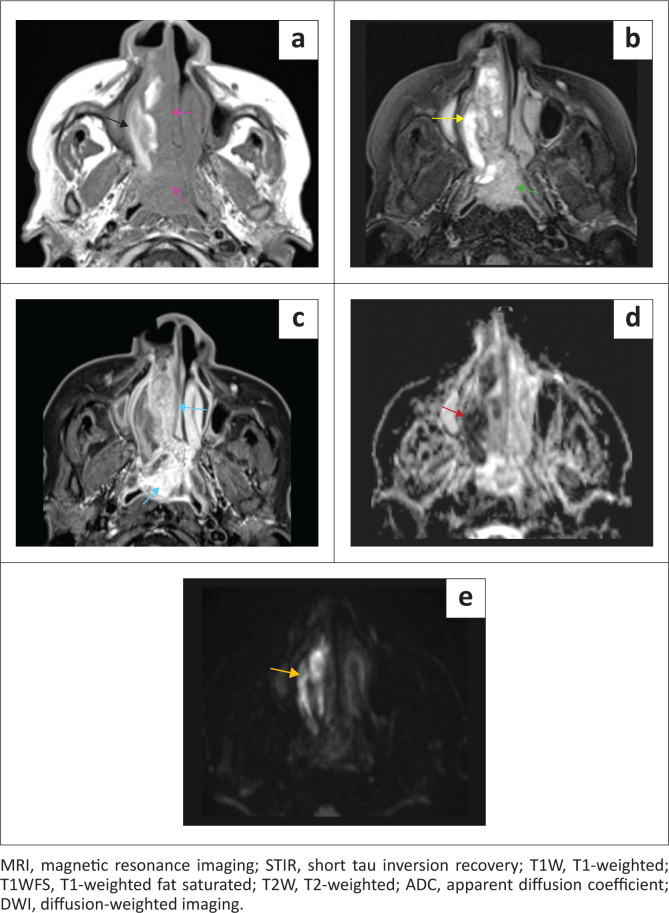
Multisequence axial MRI images (a) T1W, (b) STIR, (c) T1WFS post contrast, (d) ADC, and (e) DWI. (a) T1W isointense (purple arrows) with a hyperintense component (black arrow). (b) T2W hyperintense (green arrow) with a markedly hyperintense component (yellow arrow). (c) Heterogeneous post-contrast enhancement (blue arrows). (d, e) T1W hyperintense component demonstrates ADC hypointensity (red arrow) and DWI hyperintensity (orange arrow).

**FIGURE 6:**
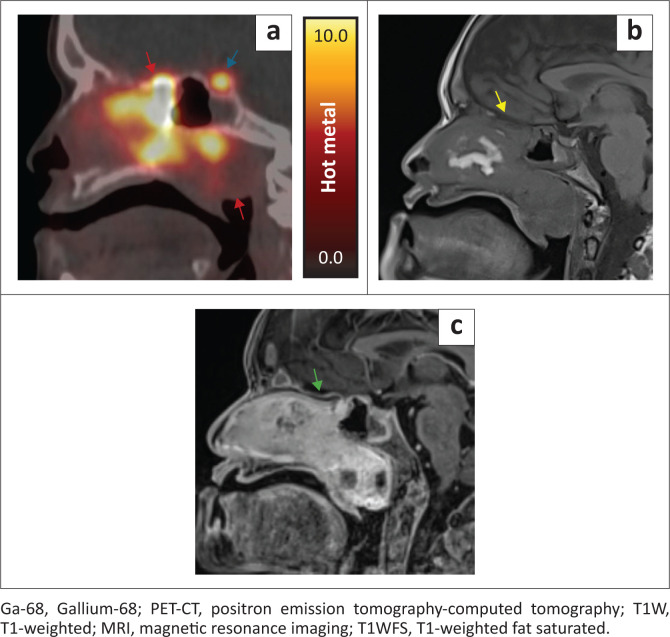
(a) Ga-68 DOTANOC PET-CT (b) T1W MRI (c) T1WFS post-contrast. Multistudy midline sagittal images through the nasal passage and nasopharynx demonstrates a mass with intense, heterogeneous DOTANOC uptake (red arrows) adjacent to an indistinct planum sphenoidale (yellow arrow) with subtle thickening and enhancement of the dura (green arrow). Physiologic pituitary DOTANOC uptake is observed (blue arrow).

There was enhancing tumour eroding the planum sphenoidale with slight thickening and enhancement of the adjacent dura consistent with dural involvement (Figure 6). The right lamina papyracea and periorbita were displaced. No surrounding soft tissue inflammatory changes were present.

A pearl and pitfall in this case is that intratumoural haemorrhage may result in photopaenia on functional imaging and pseudo-restricted diffusion on MRI. Combining the interpretation of findings and meticulously scrutinising signal characteristics of all modalities can avoid this pitfall.

### Surgery

Pre-operative areas of concern were the defects in the fovea ethmoidalis, posterior wall of the sphenoid, and the absent lamina papyracea on the right. An attempt to cauterise the anterior and posterior ethmoid arteries through a pre-caruncular approach was abandoned at the start of surgery because of the absent lamina papyracea and distorted anatomy. The tumour was resected using a four-hand endoscopic approach after all the appropriate measures were taken. The spheno-palatine artery was cauterised intra-operatively to reduce the bleeding. The bulk of the tumour was in the posterior ethmoid and sphenoid sinuses. Removing the tumour from the defect in the posterior sphenoid sinus produced profuse bleeding from the dural veins. This was controlled by manual compression and applying Surgiflo^®^. Complete macroscopic tumour resection was achieved. A defect in the fovea ethmoidalis, causing cerebrospinal fluid leakage, was repaired using fat and a posterior septal flap. Gelfoam^®^ was placed to support this, and the nose was packed with bismuth iodoform paraffin paste (BIPP) gauze. The patient had an uneventful post-operative recovery.

### Histology

**FIGURE 7:**
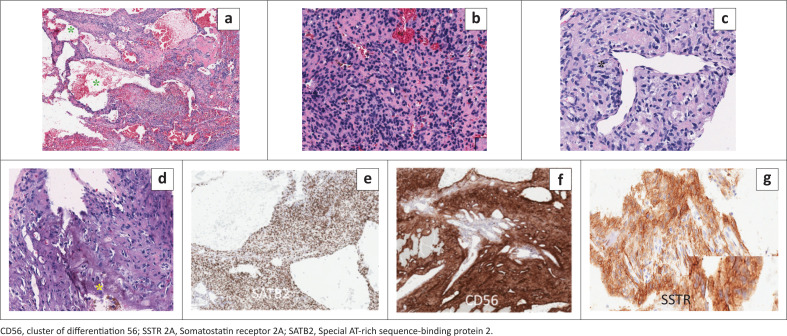
(a) Ethmoid tumour composed of microcystic spaces (green asterisks) (20x magnification). (b and c) Bland proliferation of spindle cells (40X) surrounding haemangiopericytomatous-like blood vessels (back asterisk). (d) Highlights the grungy smudgy basophilic calcifications (yellow asterisk). (e and f) Highlights SATB2 and CD56 positivity in tumour cells. (g) Cytoplasmic positivity with SSTR 2A.

Histological analysis revealed multicystic spaces overlaid with respiratory epithelium, accompanied by a proliferation of spindle cells arranged in short fascicles, creating areas of both heightened and reduced cellularity. Prominent gritty calcifications were observed, alongside perivascular hyalinisation and the condensation of spindle cells around vessels resembling those found in haemangiopericytoma. Notably, there were no signs of pleomorphism, mitotic activity or necrosis. Immunoreactivity was observed in tumour cells for SATB2, CD56, ERG, and somatostatin receptor 2A (SSTR 2A) (see Figure 7). Considering the clinical data alongside the aforementioned morphological characteristics and immunoprofile, the diagnosis of PMT was confirmed.

### Outcome

Post-operatively, oral calcium and phosphate replacement was continued initially because of hungry bone syndrome, but then stopped. Biochemical response showed normalisation of serum inorganic phosphate levels to 0.86 mmol/L, 10 days post-surgery.

The patient reports general improvement in well-being with a dramatic reduction in her chronic pain. Post-operative radiographs showed improvement in the bone mineral density and interval osseous bridging of the femoral and phalangeal fractures. Follow-up Ga-68 DOTANOC PET-CT 6 months post-surgery revealed no residual tumour.

## Discussion

The clinical presentation and biochemical workup of TIO secondary to PMT is fascinating. Although numerous case reports and articles have detailed the biochemical and histopathological findings, few have highlighted the imaging characteristics and the role of the diagnostic radiologist and nuclear physician in the diagnosis. To the authors’ knowledge, this is the first multimodality description of a sinonasal PMT from sub-Saharan Africa, comprising radiography, CT, PET-CT, and MRI, with histological confirmation.

Patients with TIO usually present with fairly non-specific symptoms. As a result of the rarity of the syndrome, this entity might not be included in the working diagnosis initially. Feng et al. reviewed 144 cases of TIO and reported an initial misdiagnosis rate of 95.1% with a tentative diagnosis of TIO often being made in excess of two and a half years of symptom onset. The average duration from the presumed diagnosis to identifying the culprit tumour is 5 years.^7^ In the presented case, there was a delay of approximately 7 years from the initial presentation and above laboratory values to definitive diagnosis. The most common reported misdiagnoses were intervertebral disk herniation, spondyloarthritis, osteoporosis and femoral head necrosis; with hyperparathyroidism, metastases, connective tissue disease and fibromyalgia syndrome being less common misdiagnoses.^7^

If TIO is suspected and a primary lesion is not clinically evident, whole body functional imaging is recommended in the tumour localisation pathway.^4,5,8^ Although these tumours are not neuroendocrine tumours, 79% of PMT’s express SSTR on their cell surface, predominantly SSTR subtype 2. Somatostatin analogues may be labelled with Gallium-68 (Ga-68 DOTANOC, DOTATOC or DOTATATE), Technetium-99m (Tc-99m-HYNIC-octreotide) and Indium-111 (Indium 111-DTPA-octreotide or Octreoscan). Ga-68-labelled SSTR analogue PET-CT is the first-line imaging modality of choice, if available,^4^ and is superior to Octreoscan and F-18-FDG PET-CT in detecting these tumours, with studies showing sensitivities ranging between 90% and 100% and specificity of 91%.^9,10^

Once the lesion is identified, anatomic imaging is needed to assist with surgical planning.^4,5,8^ A literature review of the imaging features of PMT revealed that the majority of lesions are isodense to soft tissue or heterogeneous on CT with varying degrees of post-contrast enhancement. Soft tissue axial and extremity tumours are often reported to contain internal osseous matrix, most frequently punctate, amorphous or ground glass matrix.^2,3,11,12,13^

The MRI tumour characteristics are T2W/FLAIR hyperintense, T1W isointense to muscle and demonstrating avid post-contrast enhancement. While smaller lesions are usually homogenous; larger lesions tend to be heterogenous.^1,2,11,14^ T2W dark internal foci were reported in 88.9% of a review of 36 PMT, consistent with vascular flow voids.^11,14^ Less commonly, lesions can be hyperintense on T1W and isointense or hypointense on T2W. Rarely, peripheral enhancement is encountered. Internal haemorrhage, fluid-fluid levels and perilesional oedema have also been reported.^11^ Stress or pathological fractures were present in 42.4% of the CT/MRI of the primary PMT tumour; in non-head and neck sites.^11^

Sinonasal PMT tumours cause local effect on adjacent structures such as expansion and remodelling or erosion, with benign regional effects much more common. The remainder of the imaging features are similar to the findings of axial and extremity PMT’s, including the presence of vascular flow voids.^2,3,8,12^ Intracranial invasion occurs rarely.^13^ The presented case had internal flow voids and intratumoural haemorrhage.

Although the histology of these tumours is usually benign, local aggressiveness must be considered, possibly causing multiple recurrences after first resection.^15^ Most cases follow a benign clinical course, with rare occurrences of malignant transformation.^16,17^

In the absence of a clinical suspicion of TIO, sinonasal PMTs have a wide differential diagnosis. Additionally, some of these tumours are completely silent and may grow unnoticed. Symptoms will appear when these lesions become large enough to produce pain or discomfort.^15^ Although PMTs are the most common cause of TIO, several benign and malignant lesions can produce FGF-23, such as odontogenic fibroma, haemangiopericytomas, giant cell tumour of tendons and, in a minority of cases, phosphaturic malignant tumours.^1,15^

With the more common locally benign imaging appearance, the differential diagnosis includes haemangiopericytoma, nerve sheath tumours and polyps. Rare aggressive sinonasal PMT’s may resemble malignant lesions such as squamous cell carcinoma, adenocarcinoma or sinonasal undifferentiated carcinoma.^18^

## Conclusion

Tumour-induced osteomalacia is a rare but often debilitating disorder. Phosphaturic mesenchymal tumours are a rare distinctive mesenchymal neoplasm with heterogeneous, but recognisable, histologic appearances. They frequently elicit a clinical paraneoplastic syndrome consisting of hypophosphataemic hyperphosphaturic osteomalacia due to tumoural secretion of FGF23. Multidisciplinary awareness of this entity and adequate clinical workup is key. Functional and anatomic imaging is usually required to localise the lesion and for adequate surgical planning.
